# Association of Genetic Polymorphisms in MicroRNAs With Type 2 Diabetes Mellitus in a Chinese Population

**DOI:** 10.3389/fendo.2020.587561

**Published:** 2021-02-08

**Authors:** Zaihan Zhu, Yanfen Zhang, Ruocen Bai, Ru Yang, Zhongyan Shan, Chunyan Ma, Jun Yang, Dandan Sun

**Affiliations:** ^1^Department of Cardiovascular Ultrasound, The First Affiliated Hospital of China Medical University, Shenyang, China; ^2^Department of Endocrinology and Metabolism, Institute of Endocrinology, Liaoning Provincial Key Laboratory of Endocrine Diseases, The First Affiliated Hospital of China Medical University, Shenyang, China

**Keywords:** microRNA, polymorphism, type 2 diabetes mellitus, insulin signaling pathway, Chinese population

## Abstract

**Introduction:**

MicroRNAs (miRNA) involved in the insulin signaling pathways deeply affect the pathogenesis of T2DM. The aim of this study was to assess the association between single nucleotide polymorphisms (SNP) of the related miRNAs (let-7f rs10877887, let-7a-1 rs13293512, miR-133a-1 rs8089787, miR-133a-2 rs13040413, and miR-27a rs895819) and susceptibility to type 2 diabetes mellitus (T2DM), and its possible mechanisms.

**Methods:**

Five SNPs in miRNAs (let-7f rs10877887, let-7a-1 rs13293512, miR-133a-1 rs8089787, miR-133a-2 rs13040413, and miR-27a rs895819) involved in the insulin signaling pathways were selected and genotyped in a case-control study that enrolled 371 T2DM patients and 381 non-diabetic controls. The individual SNP association analyses, interaction analyses of SNP-SNP, SNP-environmental factors were performed. The effect the risk-associated polymorphism on regulating its mature miRNA expression was also evaluated.

**Results:**

In overall analyses, miR-133a-2 rs13040413 and let-7a-1 rs13293512 were related to the susceptibility to T2DM. In stratified analyses, miR-133a-2 rs13040413, let-7a-1 rs13293512 and miR-27a rs895819 showed associations with T2DM in the age ≥ 60 years subgroup. Moreover, let-7a-1 rs13293512 and miR-27a rs895819 showed associations with T2DM in male subgroup. In SNP-environmental factors interaction analyses, there were interaction effects of miR-133a-2 rs13040413 with dyslipidemia, let-7a-1 rs13293512 with smoking, and let-7a-1 rs13293512 with dyslipidemia on T2DM. In SNP-SNP interaction analyses, there were also interaction effects of miR-133a-1 rs8089787 with let-7a-1 rs13293512, and miR-133a-1 rs8089787 with let-7f rs10877887 on T2DM. Furthermore, for miR-133a-2 rs13040413, the variant T allele showed a trend toward decreased miR-133a expression in comparison with the wild C allele. For let-7a-1 rs13293512, the variant C allele expressed a lower let-7a compared to the wild T allele.

**Conclusion:**

MiRNAs polymorphisms involved in the insulin signaling pathways and the interaction effects of SNP-SNP, SNP-environmental factors were related to T2DM susceptibility in a Chinese population.

## Introduction

Type 2 diabetes mellitus (T2DM) posting one of the most common and serious chronic diseases, is characterized sustained hyperglycemia (1). Insulin resistance, involving a defect in insulin secretion, insulin action, or both, is central to the etiology of T2DM (2). The underlying molecular mechanism for insulin resistance is only partially understood. Nevertheless, increasing evidences have showed that various microRNAs (miRNA) involved in regulating the main protein cascades in the insulin signaling pathways that affect insulin resistance, and therefore, the pathogenesis of T2DM, such as let-7f with insulin growth factor-1 receptor (IGF1R), let-7a with phosphatidylinositol 3-kinase (PI3K)/protein kinase B (AKT), miR-133a with glucose transporter 4 (GLUT4), and miR-27a with mammalian target of rapamycin (mTOR) (3–6).

It has been showed that IGF1R was the target of let-7f. Let-7f mimics could suppress IGF1R expression, and that let-7f inhibitors could increase the expression level of IGF1R (3). Blood level of let-7f was down-regulated in T2DM subjects compared to controls (7). Moreover, let-7a is associated with PI3K/AKT signaling. Let-7a overexpression could decrease the expression levels of PI3K and p-AKT (4). Down-regulation of let-7a level was observed in T2DM subjects (8). Overexpression of miR-133a could decrease GLUT4 expression and reduced insulin-mediated glucose uptake (5). The circulatory miR-133a level was significantly higher in T2DM subjects than in controls. Further, there was a positive and significant correlation between miR-133a with fasting blood glucose (FBG) and glycated hemoglobin in the T2DM subjects (9). Elevated miR-27a could up-regulate mTOR phosphorylation level and enhance mTOR signaling (6). Increased blood level of miR-27a was identified in T2DM, and associated with measures of pancreatic β cell function (10). Collectively, let-7f, let-7a, miR-133a, and miR-27a have strong relationship with T2DM, both in underlying mechanisms and expression levels.

Single nucleotide polymorphisms (SNP) are variations in single nucleotides of genomic DNA sequence, which have modified potentials on gene functions (11). SNPs affect the miRNA binding efficiency, giving rise to increased or decreased miRNA regulation (12). We hypothesize that the SNPs in miRNA may impact the susceptibility to T2DM by miRNA dysregulation of mRNA degradation, translation, and expression. It has been showed that let-7f rs10877887 and let-7a-1 rs13293512 have relationships with stroke, depression, aneurysms and cancers (13–16). Especially, let-7a-1 rs13293512 have binding sites with interleukin-6 (IL-6), a contributor of T2DM. The variant genotypes of let-7a-1 rs13293512 were associated with increased IL-6 expression (17). MiR-133a-1 rs8089787 are associated with asthma (18). MiR-133a-2 rs13040413 variant could increase levels of miR-133a (19). However, there was no study concerning about the associations between let-7f rs10877887, let-7a-1 rs13293512, miR-133a-1 rs8089787 and miR-133a-2 rs13040413 and T2DM. Furthermore, the relationships of miR-27a rs895819 and T2DM were controversial and inconclusive, which need further investigation (20). In addition, only a part of T2DM heritability can be interpreted by a single miRNA polymorphism study. SNP-SNP and SNP-environmental factors interactions may account for another missing part of the heritability. But there are lacking studies on the interactions of miRNA SNPs, as well as with environmental factors in T2DM.

Thus, the aim of our study is comprehensively investigating the effects of variations in miRNA genes involving in insulin signaling pathways, let-7f rs10877887, let-7a-1 rs13293512, miR-133a-1 rs8089787, miR-133a-2 rs13040413, and miR-27a rs895819, the interactions of SNP-SNP, SNP-environmental factors on T2DM in a Chinese population, as well as the effect of the risk-associated polymorphism on regulating its mature miRNA expression.

## Materials and Methods

### Study Subjects

A total of 752 participants were enrolled in this study, including 371 untreated T2DM patients and 381 non-diabetic controls. T2DM participants were recruited from inpatient and outpatient services at First Affiliated Hospital of China Medical University, and control participants from local community between August 2016 and December 2018. T2DM met the 1999 WHO criteria for diabetes: a FBG ≥ 7.0 mmol/l or a 2h blood glucose (2hBG) ≥ 11.1mmol/l (21). The non-diabetic controls were included as follows: a fasting glucose level < 6.1 mmol/l or 2h glucose level < 7.8 mmol/l, no past history of diabetes and no family history of T2DM. Exclusion criteria were participants with type 1 diabetes or other special types of diabetes, malignant disease, liver and kidney disease, acute infections, or myocardial infarction. All participants provided written informed consent after receiving a detailed description of the study. The study was approved by the Institutional Ethics Committee of China Medical University. This study was carried out in accordance with the standard biosecurity and institutional safety procedures of China Medical University.

### Data Collection, Anthropometric Measure, and Laboratory Testing

Data of sex, age, body mass index (BMI), smoking status, and alcohol consumption were collected from questionnaires. Smokers were defined as having smoked at least one cigarette per day for more than one year. Drinkers were defined as having consumed at least one alcoholic beverage a day for a minimum period of six months. Systolic blood pressure (SBP), diastolic blood pressure (DBP), blood glucose, total cholesterol (TC), high-density lipoprotein cholesterol (HDL), low-density lipoprotein cholesterol (LDL), and triglyceride (TG) were measured using standard laboratory procedures. Hypertension was defined as ≥ 140/90 mmHg or any antihypertensive treatment. Dyslipidemia was defined as TC ≥ 5.17 mmol/L, or TG ≥ 1.70 mmol/L, or LDL ≥ 2.58 mmol/L, or HDL ≤ 0.91 mmol/L or under taking hypolipidemic drugs.

### SNP Selection and Genotyping

Five tag-SNPs were identified as the following steps (22). First, the candidate SNPs were screened in the 5′ and 3′ region, mature sequence, pri-miRNA sequence, pre-miRNA sequence of the miRNA genes. Second, the candidate SNPs were selected in the combinations provided by the HapMap database (http://www.HapMap.org) and Haploview software 4.0 (http://www.broadinstitute.org/mpg/haploview). Third, the potential functions of the candidate SNPs were predict by the web-based analysis tools (SNPinfo, http://snpinfo.niehs.nih.gov; PolymiRTS Database 3.0, http://compbio.uthsc.edu; TFSEARCH 1.3, http://www.cbrc.jp/research/db/TFSEARCH.html). Collectively, miR-133a-1 rs8089787, miR-133a-2 rs13040413, let-7a-1 rs13293512, let-7f rs10877887, and miR-27a rs895819 were selected for genotyping.

Genomic DNA was extracted using the standard phenol-chloroform method, and then diluted to a working concentration of 50ng/μl before genotyping (23). The assays, primer design, and genotyping of candidate gene polymorphisms were performed by Baygene Biotechnology Company Limited (Shanghai, China) using the KASP method with SNPLine platform (LGC, United Kingdom). For genotyping quality controlling, 5% samples were repeated genotyped and the results were 100% consistent.

### Transfection and Real-Time PCR Reaction for miRNA Expression

The expression vectors pGCMV-rs13040413-C, pGCMV-rs13040413-T (site-specific mutagenesis from C to T), pGCMV-rs13293512-T, and pGCMV-rs13293512-C (site-specific mutagenesis from T to C) were synthesized by Genechem Company (Genechem Co. Ltd, Shanghai, China). The pGCMV-rs13040413-C, pGCMV-rs13040413-T, pGCMV-rs13293512-T, pGCMV-rs13293512-C, and empty plasmids were transfected into human embryonic kidney (HEK) 293T cells, respectively. After 48 h, the total RNA was extracted using TRIzol reagent (Invitrogen, USA). Real-time PCR was used to detect miR-133a and let-7a expression using TaqMan microRNA assay kits (ABI, USA). All experiments were carried out in triplicate.

### Statistical Analysis

All statistical analyses were carried out with SPSS 16.0 software (SPSS, Chicago, IL, USA). Continuous variables were presented as mean ± SD and compared by Student’s t test, and discrete variables, including the Hardy-Weinberg equilibrium (HWE) in control group, as frequencies and percentages and by Chi-square χ2 test. The association of SNPs and T2DM risk was assessed by the odds ratios (OR) with 95% confidence intervals (CI) using multiple logistic regression analysis after adjustment for potential risk factors (age, gender, smoking, drinking, hypertension, and dyslipidemia). The interaction effects (SNP-SNP, SNP-environment) were evaluated by the likelihood-ratio with a fully parameterized model. Differential expression of the wild type and mutant alleles was analyzed by Student’s t test. A two-side *P*-value less than 0.05 was considered statistically significant.

## Results

### Demographic and Clinical Characteristics

Demographic and clinical data are presented in **Table 1**. There were no significant differences in sex, age, BMI, smoking, and drinking between T2DM patients and controls (*P* > 0.05). T2DM patients had significant higher FBG, 2hBG, SBP, DBP, and TG, and lower HDL when compared with controls (*P* < 0.05). The dyslipidemia rate was higher in T2DM patients compared to controls (*P* < 0.05).

**Table 1:** Demographics and clinical characteristics of type 2 diabetes mellitus cases and non-diabetic controls.

Variables	T2DM (N=371)	controls (N=381)	T/χ2	*P* value
Sex, M/F (%)	196/175 (52.8/47.2)	192/189 (50.4/49.6)	0.447	0.504
Age (years)	54.15 ± 10.55	54.71 ± 8.96	-0.778	0.437
Body mass index (kg/m2)	25.35 ± 3.74	25.38 ± 3.28	-0.070	0.944
Smoking, Y/N (%)	113/258 (30.4/69.6)	98/283 (25.5/74.5)	3.463	0.073
Drinking, Y/N (%)	75/296 (20.0/80.0)	68/313 (17.6/82.4)	0.689	0.407
Fasting blood glucose (mmol/L)	8.48 ± 2.77	5.34 ± 0.55	21.591	<0.001
2h blood glucose (mmol/L)	15.34 ± 5.29	6.29 ± 1.92	29.007	<0.001
Systolic blood pressure (mmHg)	138.301 ± 21.07	133.85 ± 19.96	-2.969	0.003
Diastolic blood pressure (mmHg)	87.49 ± 13.07	82.82 ± 13.27	-4.865	<0.001
Total cholesterol (mmol/L)	5.08 ± 1.15	5.03 ± 2.76	-0.332	0.740
Triglyceride (mmol/L)	2.33 ± 2.36	1.44 ± 1.03	6.659	<0.001
Low-density lipoprotein cholesterol (mmol/L)	3.20 ± 0.90	3.17 ± 0.96	-0.530	0.596
High-density lipoprotein cholesterol (mmol/L)	1.24 ± 0.54	1.40 ± 0.67	-3.452	0.001
Hypertension, Y/N (%)	157/214 (42.3/57.7)	140/241 (36.7/63.3)	2.466	0.116
Dyslipidemia, Y/N (%)	235/136 (63.5/36.5)	201/180 (52.8/47.2)	8.921	0.003

T2DM, type 2 diabetes mellitus.

### Association of MiRNA Polymorphisms With T2DM

All genotypes were distributed in accordance with Hardy–Weinberg equilibrium (*P* > 0.05). As shown in **Table 2**, miR-133a-2 rs13040413 variant genotypes were significantly associated with an increased risk of T2DM (TT: OR = 2.18, *P* = 0.028; CT+TT: OR = 1.38, *P* = 0.040). let-7a-1 rs13293512 variant genotypes were also associated with an increased risk of T2MD (CC: OR = 1.61, *P* = 0.026; TC+CC: OR = 1.41, *P* = 0.029). There was no overall genetic effect on T2DM for miR-133a-1 rs8089787, let-7f rs10877887, and miR-27a rs895819 (*P* > 0.05).

**Table 2:** Association of miRNA polymorphisms with type 2 diabetes mellitus.

Genotype			Crude models		Adjusted models	
			OR(95%CI)	*P* value	OR(95%CI)^a^	*P* value
miR-133a-1 rs8089787						
CT	vs	CC	0.90(0.63, 1.27)	0.540	0.91(0.64, 1.29)	0.583
TT	vs	CC	1.00(0.32, 3.15)	0.995	1.05(0.33, 3.32)	0.929
CT+TT	vs	CC	0.90(0.64, 1.27)	0.558	0.91(0.65, 1.29)	0.603
miR-133a-2 rs13040413						
CT	vs	CC	1.26(0.92, 1.75)	0.152	1.27(0.92, 1.76)	0.145
TT	vs	CC	2.19(1.10, 4.38)	0.026	2.18(1.09, 4.36)	0.028
CT+TT	vs	CC	1.38(1.01, 1.87)	0.041	1.38(1.02, 1.87)	0.040
let-7a-1 rs13293512						
TC	vs	TT	1.35(0.97, 1.88)	0.074	1.34(0.97, 1.88)	0.080
CC	vs	TT	1.61(1.06, 2.44)	0.026	1.61(1.06, 2.44)	0.026
TC+CC	vs	TT	1.42(1.04, 1.93)	0.028	1.41(1.04, 1.93)	0.029
let-7f rs10877887						
TC	vs	TT	1.22(0.90, 1.66)	0.197	1.21(0.89, 1.65)	0.219
CC	vs	TT	0.83(0.52, 1.31)	0.414	0.82(0.52, 1.30)	0.404
TC+CC	vs	TT	1.12(0.84, 1.49)	0.442	1.11(0.83, 1.48)	0.467
miR-27a rs895819						
AG	vs	AA	1.26(0.93, 1.71)	0.138	1.26(0.93, 1.71)	0.134
GG	vs	AA	1.27(0.72, 2.23)	0.408	1.26(0.72, 2.22)	0.421
AG+GG	vs	AA	1.26(0.94, 1.68)	0.116	1.26(0.94, 1.68)	0.117

^a^ORs and 95%CI and corresponding P values were calculated by logistic regression analysis adjusted by sex, age, smoking, drinking, hypertension, and dyslipidemia; OR, odds ratio; CI, confidence interval.

Moreover, stratified analyses were conducted to evaluate the associations between SNPs and T2DM by age and sex (**Table 3**). miR-133a-2 rs13040413 variant genotype was associated with an increased risk of T2MD in the age ≥ 60 years subgroup (TT: OR = 5.42, *P* = 0.009). let-7a-1 rs13293512 variant genotypes were associated with an increased risk of T2MD in both the age ≥ 60 years (TC: OR = 2.19, *P* = 0.010; CC: OR = 2.40, *P* = 0.005; TC+CC: OR = 3.20, *P* = 0.002) and male subgroup (CC: OR = 1.87, *P* = 0.040). miR-27a rs895819 variant genotypes were also associated with an increased risk of T2MD in both the age ≥ 60 years (GG: OR = 4.92, *P* = 0.049) and male subgroup (AG: OR = 1.53, *P* = 0.049; TC+CC: OR = 1.59, *P* = 0.039). No statistical significant differences were observed between miR-133a-1 rs8089787 or let-7f rs10877887 polymorphisms and T2DM (*P* > 0.05).

**Table 3:** Stratified analysis of association between miRNA polymorphisms and type 2 diabetes mellitus.

Genotype			Male		Female		≥60		<60	
			OR(95%CI)^a^	*P* value	OR(95%CI)^a^	*P* value	OR(95%CI)^b^	*P* value	OR(95%CI)^b^	*P* value
miR-133a-1 rs8089787										
CT	vs	CC	0.76(0.45, 1.27)	0.289	1.05(0.65, 1.70)	0.850	1.31(0.70, 2.44)	0.402	0.74(0.48, 1.14)	0.165
TT	vs	CC	0.46(0.04, 5.07)	0.522	1.36(0.36, 5.19)	0.651	1.87(0.16, 21.35)	0.616	0.86(0.23, 3.26)	0.822
CT+TT	vs	CC	0.74(0.44, 1.23)	0.248	1.07(0.67, 1.71)	0.767	1.33(0.72, 2.45)	0.367	0.74(0.49, 1.13)	0.167
miR-133a-2 rs13040413										
CT	vs	CC	1.29(0.82, 2.02)	0.274	1.21(0.76, 1.95)	0.424	1.26(0.79, 2.010)	0.336	1.20(0.76, 1.89)	0.436
TT	vs	CC	2.27(0.76, 6.76)	0.141	2.13(0.86, 5.27)	0.103	5.42(1.52, 19.30)	0.009	1.22(0.49, 3.05)	0.672
CT+TT	vs	CC	1.38(0.90, 2.13)	0.145	1.31(0.85, 2.04)	0.226	1.50(0.96, 2.33)	0.077	1.19(0.78, 1.83)	0.425
let-7a-1 rs13293512										
TC	vs	TT	1.34(0.85, 2.11)	0.213	1.35(0.84, 2.17)	0.219	2.19(1.21, 3.97)	0.010	1.08(0.72, 1.60)	0.714
CC	vs	TT	1.87(1.03, 3.39)	0.040	1.38(0.76, 2.50)	0.287	2.40(1.37, 4.23)	0.005	1.25(0.76, 2.05)	0.375
TC+CC	vs	TT	1.47(0.95, 2.26)	0.084	1.36(0.87, 2.13)	0.179	3.20(1.42, 7.22)	0.002	1.12(0.77, 1.64)	0.545
let-7f rs10877887										
TC	vs	TT	1.17(0.77, 1.80)	0.466	1.24(0.80, 1.93)	0.336	1.44(0.82, 2.51)	0.200	1.14(0.79, 1.65)	0.486
CC	vs	TT	0.70(0.36, 1.36)	0.290	1.01(0.52, 1.94)	0.988	0.99(0.40, 2.46)	0.986	0.82(0.48, 1.41)	0.472
TC+CC	vs	TT	1.05(0.70, 1.57)	0.808	1.18(0.78, 1.79)	0.429	1.34(0.79, 2.27)	0.282	1.05(0.75, 1.49)	0.764
miR-27a rs895819										
AG	vs	AA	1.53(1.00, 2.34)	0.049	1.02(0.66, 1.58)	0.925	1.35(0.78, 2.34)	0.289	1.23(0.85, 1.77)	0.267
GG	vs	AA	1.50(0.69, 3.25)	0.308	1.01(0.44, 2.33)	0.977	4.92(1.01, 24.05)	0.049	0.95(0.50, 1.80)	0.874
AG+GG	vs	AA	1.59(1.07, 2.59)	0.039	1.02(0.67, 1.54)	0.942	1.53(0.90, 2.61)	0.119	1.17(0.83, 1.66)	0.366

^a^ORs and 95%CI and corresponding P values were calculated by logistic regression analysis adjusted by age, smoking, drinking, hypertension, and dyslipidemia; ^b^ORs and 95%CI and corresponding P values were calculated by logistic regression analysis adjusted by sex, smoking, drinking, hypertension, and dyslipidemia; OR, odds ratio; CI, confidence interval.

### Interactions of MiRNA Polymorphisms and Environmental Factors in T2DM

Interaction effects of miRNA polymorphisms and environmental factors in T2DM were also explored. The models included SNPs with smoking, drinking, hypertension, and dyslipidemia. As presented in **Table 4**, miR-133a-2 rs13040413 variant genotype had a positive interaction with dyslipidemia on T2DM (OR = 10.26, *P* = 0.004). let-7a-1 rs13293512 variant genotypes also had positive interactions with smoking (TC: OR = 2.04, *P* = 0.049; TC+CC: OR = 2.15, *P* = 0.027) and dyslipidemia (CC: OR = 1.93, *P* = 0.040; TC+CC: OR = 2.56, *P* = 0.030) on T2DM. However, there were no significant differences among other gene polymorphisms and environmental factors interactions (*P* > 0.05).

**Table 4:** SNP-environmental factors interaction effects on type 2 diabetes mellitus.

			Smoking (+) vs (-)		Drinking (+) vs (-)		Hypertension (+) vs (-)		Dyslipidemia (+) vs (-)	
			OR(95%CI)^a^	*P* value	OR(95%CI)^b^	*P* value	OR(95%CI)^c^	*P* value	OR(95%CI)^d^	*P* value
miR-133a-1 rs8089787										
CT	vs	CC	1.26(0.63, 2.56)	0.514	0.78(0.36, 1.68)	0.524	1.08(0.47, 2.49)	0.857	0.73(0.26, 2.04)	0.550
TT	vs	CC	4.77(0.32, 70.74)	0.257	1.05(0.33, 3.30)	0.935	1.87(0.12, 29.59)	0.656	1.05(0.33, 3.30)	0.935
CT+TT	vs	CC	1.36(0.68, 2.69)	0.385	0.89(0.42, 1.89)	0.756	1.13(0.50, 2.53)	0.775	0.73(0.26, 2.01)	0.538
miR-133a-2 rs13040413										
CT	vs	CC	1.02(0.54, 1.96)	0.946	1.42(0.70, 2.90)	0.333	1.82(0.86, 3.88)	0.120	0.87(0.38, 1.96)	0.730
TT	vs	CC	2.24(0.38, 13.14)	0.371	1.87(0.95, 3.67)	0.068	2.24(0.38, 13.14)	0.371	1.08(0.21, 5.46)	0.929
CT+TT	vs	CC	1.24(0.67, 2.29)	0.491	10.26(2.08, 50.56)	0.004	1.86(0.91, 3.82)	0.089	0.92(0.43, 1.97)	0.824
let-7a-1 rs13293512										
TC	vs	TT	2.04(1.00, 4.17)	0.049	1.75(0.91, 3.39)	0.096	1.06(0.51, 2.20)	0.882	0.74(0.32, 1.75)	0.498
CC	vs	TT	2.58(1.00, 6.68)	0.051	1.93(1.03, 3.60)	0.040	0.67(0.27, 1.66)	0.382	1.00(0.33, 3.04)	0.997
TC+CC	vs	TT	2.15(1.09, 4.23)	0.027	2.56(1.09, 6.01)	0.030	0.93(0.47, 1.84)	0.828	0.80(0.35, 1.80)	0.583
let-7f rs10877887										
TC	vs	TT	1.40(0.76, 2.58)	0.286	1.50(0.76, 2.97)	0.240	0.81(0.41, 1.61)	0.551	0.55(0.25, 1.21)	0.136
CC	vs	TT	1.74(0.69, 4.42)	0.241	2.33(0.81, 6.69)	0.116	1.11(0.38, 3.21)	0.850	1.46(0.46, 4.66)	0.520
TC+CC	vs	TT	1.46(0.82, 2.59)	0.202	1.64(0.86, 3.11)	0.134	0.88(0.46, 1.67)	0.689	0.69(0.33, 1.43)	0.318
miR-27a rs895819										
AG	vs	AA	1.11(0.61, 2.05)	0.730	0.97(0.49, 1.91)	0.926	1.18(0.59, 2.34)	0.640	1.30(0.59, 2.84)	0.518
GG	vs	AA	2.38(0.74, 7.67)	0.147	0.77(0.21, 2.81)	0.695	2.86(0.79, 10.43)	0.111	0.79(0.20, 3.12)	0.738
AG+GG	vs	AA	1.26(0.70, 2.24)	0.442	0.93(0.49, 1.78)	0.833	1.37(0.72, 2.63)	0.340	1.18(0.57, 2.48)	0.656

^a^ORs and 95%CI and corresponding P values were calculated by logistic regression analysis adjusted by age, sex, drinking, hypertension, and dyslipidemia; b, ORs and 95%CI and corresponding P values were calculated by logistic regression analysis adjusted by age, sex, smoking, hypertension, and dyslipidemia; ^c^ORs and 95%CI and corresponding P values were calculated by logistic regression analysis adjusted by age, sex, smoking, drinking, and dyslipidemia; ^d^ ORs and 95%CI and corresponding P values were calculated by logistic regression analysis adjusted by age, sex, smoking, drinking, and hypertension; SNP, single nucleotide polymorphism; OR, odds ratio; CI, confidence interval.

### MiRNA SNP-SNP Interactions in T2DM

We further investigated miRNA SNP-SNP interaction effects in T2DM. The models included dominant genotypes of miRNA polymorphisms. It was showed that miR-133a-1 rs8089787 variant genotype and let-7a-1 rs13293512 variant genotype had a positive interaction effect in T2DM (OR = 3.79, *P* = 0.037). Moreover, there was a positive interaction effect of miR-133a-1 rs8089787 variant genotype and let-7f rs10877887 variant genotype in T2DM (OR = 3.61, P = 0.035) (**Table 5**). No significant differences were observed among other miRNA SNP-SNP interactions (*P* > 0.05).

**Table 5:** SNP-SNP interaction effects on type 2 diabetes mellitus.

Genetic model			OR(95%CI)^a^	*P* value
miR-133a-2 rs13040413	×	miR-133a-1 rs8089787	0.78(0.38, 1.62)	0.510
CT+TT vs CC		CT+TT vs CC		
miR-133a-2 rs13040413	×	let-7a-1 rs13293512	1.00(0.51, 1.95)	0.997
CT+TT vs CC		TC+CC vs TT		
miR-133a-2 rs13040413	×	let-7f rs10877887	1.42(0.77, 2.63)	0.265
CT+TT vs CC		TC+CC vs TT		
miR-133a-2 rs13040413	×	miR-27a rs895819	0.84(0.45, 1.56)	0.581
CT+TT vs CC		AG+GG vs AA		
miR-133a-1 rs8089787	×	let-7a-1 rs13293512	3.79(1.60, 8.41)	0.037
CT+TT vs CC		TC+CC vs TT		
miR-133a-1 rs8089787	×	let-7f rs10877887	3.61(2.01, 5.38)	0.035
CT+TT vs CC		TC+CC vs TT		
miR-133a-1 rs8089787	×	miR-27a rs895819	1.07(0.54, 2.13)	0.844
CT+TT vs CC		AG+GG vs AA		
let-7a-1 rs13293512	×	let-7f rs10877887	0.87(0.46, 1.62)	0.654
TC+CC vs TT		TC+CC vs TT		
let-7a-1 rs13293512	×	miR-27a rs895819	0.78(0.42, 1.47)	0.443
TC+CC vs TT		AG+GG vs AA		
let-7f rs10877887	×	miR-27a rs895819	1.25(0.70, 2.22)	0.458
TC+CC vs TT		AG+GG vs AA		

^a^ORs and 95%CI and corresponding P values were calculated by logistic regression analysis adjusted by age, sex, smoking, drinking, hypertension, and dyslipidemia; SNP, single nucleotide polymorphism; OR, odds ratio; CI, confidence interval.

### The Effect of rs13040413 and rs13293512 on miR-133a and let-7a Expression

**Figure 1:**
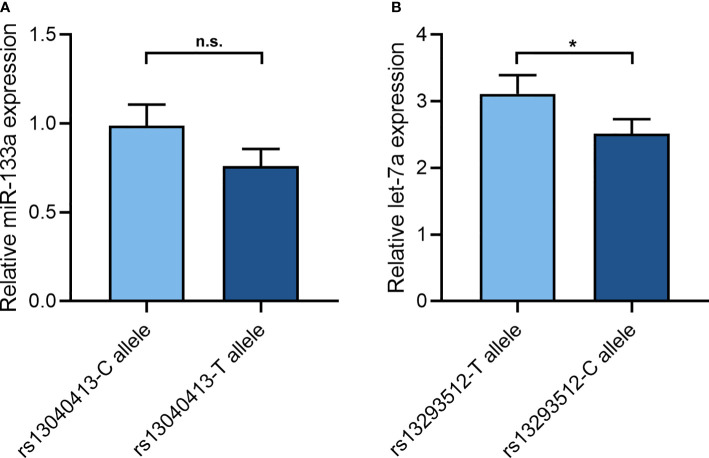
The effect of rs13040413 and rs13293512 on miR-133a and let-7a expression. **(A)** The cell miR-133a expression by different miR-133a-2 rs13040413 plasmids. **(B)** The cell let-7a expression by different let-7a-1 rs13293512 plasmids. *P < 0.05. n.s., no significance.

After 48 h, let-7a expression had statistical significance in HEK293T cell lines (**Figure 1B**). The variant C allele expressed a lower let-7a when compared to the wild T allele (2.51 ± 0.22 vs 3.11 ± 0.28, P = 0.047). While miR-133a expression did not reach statistical significance in HEK293T cell lines (**Figure 1A**). The variant T allele showed a trend toward decreased miR-133a expression in comparison with the wild C allele (0.76 ± 0.10 vs 0.99 ± 0.12, P = 0.065).

## Discussion

Alterations in the miRNA involved in the insulin signaling pathways play important roles in the pathogenesis of T2DM. SNPs in these miRNA may down-regulate or up-regulate miRNA expression in T2DM (24). The present study focused on the relation of SNPs of miRNA involved in the insulin signaling pathways, miR-133a-1 rs8089787, miR-133a-2 rs13040413, let-7a-1 rs13293512, let-7f rs10877887, and miR-27a rs895819, with T2DM, and the interaction effects of SNP-SNP and SNP-environmental factors on T2DM, as well as the effect of the risk-associated polymorphism on regulating its mature miRNA expression. To our knowledge, this is the first to comprehensively report the issues.

Our findings revealed that miR-133a-2 rs13040413 and let-7a-1 rs13293512 were associated with increased T2DM risk. For miR-133a-2 rs13040413, the variant T allele showed a trend toward decreased miR-133a expression in comparison with the wild C allele. For let-7a-1 rs13293512, the variant C allele expressed a lower let-7a compared to the wild T allele. It has been suggested that miR-133a can affect IGF1R and GLUT4 expression in the insulin signaling pathways. Gong et al. verified that IGF1R gene is a direct target of miR-133a using luciferase reporter assays (25). MiR-133a can directly suppress the expression of IGF1R through translational repression. Moreover, miR-133a has been reported to act indirectly upon GLUT4 expression. MiR-133a can target the Krueppel-like factor 15 (Klf15) mRNA, inhibiting this transcriptional factor, which is an enhancer of GLUT4 expression, thus leading to the reduction in GLUT4 expression and in insulin stimulated glucose uptake (5). MiR-133a not only showed differential expression in blood level of T2DM, but also in tissue levels of T2DM complications, such as kidney, heart, sciatic nerve, and skeletal muscle (26–29). MiR-133a-2 rs13040413 variant could also increase levels of miR-133a (19). All the above contribute to the underlying mechanisms of the association between miR-133a-2 rs13040413 and T2DM. With regard to let-7a-1 rs13293512, it has been showed that the targets of let-7a is IGF1R, insulin receptor substrate 2 (IRS2) and PI3K/AKT of insulin signaling pathways. Exogenous expression of let−7a can suppress the expression of IGF1R (30). Meanwhile, inhibition of let-7a is sufficient to enhance the expression of IRS2 (31). Wang et al. has also showed that transfection of let−7a mimics can lead to the inhibition of the PI3K/AKT, and transfection of let−7a inhibitors may activate the PI3K/AKT through the increase in PI3K and AKT levels (4). IGF1R, IRS2, and PI3K/AKT are known to mediate both insulin signaling pathways and insulin-induced cell dysregulations. As a functional polymorphism, let-7a-1 rs13293512 can substantially reduce the transcription activity of let-7, as well as directly upregulate IL-6 expression (17). The deep understanding of the potential mechanisms will yield further insights into the relationship of let-7a-1 rs13293512 and T2DM.

Additionally, in the stratified analyses, miR-133a-2 rs13040413, let-7a-1 rs13293512, and miR-27a rs895819 were associated with increased T2DM risk in older subjects (age ≥ 60 years), suggesting that age effect was dominant cause of T2DM in older subjects. Accumulated exposure to insulin disturbance caused by these three SNPs and weak immune system were expected in older subjects with T2DM. Let-7a-1 rs13293512 and miR-27a rs895819 were associated with increased T2DM risk in male subjects. Hormonal differences between males and females may elucidate sex-specific variation in T2DM. There is evidence to show bidirectional signaling cross-talk between let-7a, miR-27a, and estrogen receptors (32, 33). A role for estrogen in the regulation of glucose metabolism provides a potential biologic explanation to our results (34).

Being a multifactorial disease, there might be complex interactions between the risk allele and confounding factors in T2DM. In our study, interaction effects on T2DM have been demonstrated between miR-133a-2 rs13040413 or let-7a-1 rs13293512 and dyslipidemia. It has been showed that the expression levels of miR-133a and let-7a were in a dysregulation pattern under high lipid condition (35). Evidence also showed that miR-133a and let-7a were also related to lipid accumulation (36, 37). Additionally, we observed there was interaction effect between let-7a-1 rs13293512 and smoking on T2DM. Banerjee A. showed that let-7a was differentially expressed between the smoking and nonsmoking subjects (38). Let-7a showed a correlation with haemoglobin adduct biomarkers of tobacco exposure. Moreover, besides miR-133a and let-7a, dyslipidemia and smoking have been known as high risk factors of T2DM. Collectively, we assumed that miR-133a and let-7a had cross-talk with dyslipidemia or smoking, which could explain the phenomenon of interaction effects between miR-133a-2 rs13040413 or let-7a-1 rs13293512 and dyslipidemia or smoking on T2DM.

In our study, we also detected interaction effects of miR-133a-1 rs8089787 and let-7a-1 rs13293512 or let-7f rs10877887 on T2DM. This phenomenon is defined as epistatic effect, which usually accounts for missing or underestimated heritability when one single gene is included in disease susceptibility (39). Likely, miR-133a rs8089787 had no effect on T2DM. In contrast, miR-133a-1 rs8089787 and let-7a-1 rs13293512 or let-7f rs10877887 worked together to generate interaction effects on T2DM. We assumed that the functional effect of miR-133a, let-7a, and let-7f in the insulin signaling pathway might account for the observed interaction effect. As described above, miR-133a and let-7a were involved in the insulin signaling pathway and the pathogenesis of T2DM. As for let-7f, it has been reported that let-7f could target multiple key genes related to insulin signaling pathway. Mellios et al. reported that let-7f could inhibit IGF1 expression, and inhibition of let-7f could significantly up-regulate levels of IGF1 mRNA (40). Hu et al. showed that let-7f mimics suppressed IGF1R expression, and let-7f inhibitors increased the expression level of IGF1R (3). Furthermore, Wang et al. revealed that insulin-like growth factor 2 mRNA binding protein 1 (IGF2BP1) was potential target of let-7f. Let-7f could suppress IGF2BP1 expression (41). Combined, any genetic mutation in the insulin signaling pathway, like miR-133a-1 rs8089787, let-7a-1 rs13293512, and let-7f rs10877887, could potentially alter the action of each other so as to influence insulin function in pathogenesis of T2DM.

Several limitations of this study should be noted. First, the sample size in this study was relatively small, which might restrict the ability to explore weak genetic effect on T2DM. Prospective studies consisting of larger-scale sample and multicenter surveys are necessary to validate the findings of miRNA polymorphisms on T2DM. Second, only miRNA polymorphisms were included in this study. Further studies should involve SNPs in the miRNA binding site of potential targeted genes, which could deeply explored the SNP-SNP interactions in miRNA regulatory pathways. Third, considering the clinical significance of the study, the more comprehensive functional and molecular experiments of the mentioned miRNA polymorphisms on T2DM would be of great significance for clinical practice and may be an important future direction.

## Conclusions

This study, for the first time, investigated the relationships of miRNA polymorphisms involving in insulin signaling pathways and T2DM, and reported that miR-133a-2 rs13040413, let-7a-1 rs13293512, and miR-27a rs895819 were related to the susceptibility to T2DM in overall or stratified analyses in a Chinese population. For miR-133a-2 rs13040413, the variant T allele showed a trend toward decreased miR-133a expression in comparison with the wild C allele. For let-7a-1 rs13293512, the variant C allele expressed a lower let-7a compared to the wild T allele. Novel interaction effects on T2DM were revealed among miR-133a-2 rs13040413 with dyslipidemia, let-7a-1 rs13293512 with smoking, let-7a-1 rs13293512 with dyslipidemia, miR-133a-1 rs8089787 with let-7a-1 rs13293512, and miR-133a-1 rs8089787 with let-7f rs10877887. Future large-scale studies and more comprehensive mechanism experiments are required.

## Data Availability Statement

The datasets presented in this study can be found in online repositories. The names of the repository/repositories and accession number(s) can be found in the article/supplementary material.

## Ethics Statement

The studies involving human participants were reviewed and approved by the Institutional Review Board of China Medical University. The patients/participants provided their written informed consent to participate in this study.

## Author Contributions

DS conceptualized and supervised the study. JY and CM designed the study together with DS, ZZ, YZ, and RB carried out genomic DNA extraction and SNP genotyping. RY and ZS participated in the clinical data and blood sample collections. DS and YZ did the statistical analyses. All authors contributed to data interpretation. DS, ZZ, and YZ wrote the paper with input from all authors. JY and CM revised the manuscript. All authors contributed to the article and approved the submitted version.

## Funding

This work was supported by research grants from the National Natural Science Foundation of China (81701703 to DS), and the Shenyang Science and Technology Plan Project (19-112-4-061 to JY).

## Conflict of Interest

The authors declare that the research was conducted in the absence of any commercial or financial relationships that could be construed as a potential conflict of interest.
